# Influence of Equatorial Co-Ligands on the Reactivity of LFe^III^OIPh

**DOI:** 10.3390/molecules29010058

**Published:** 2023-12-21

**Authors:** Dóra Lakk-Bogáth, Dénes Pintarics, Patrik Török, József Kaizer

**Affiliations:** Research Group of Bioorganic and Biocoordination Chemistry, University of Pannonia, H-8201 Veszprém, Hungary; lakkd@almos.uni-pannon.hu (D.L.-B.); pintarics.denes@gmail.com (D.P.); patriktrk6@gmail.com (P.T.)

**Keywords:** non-heme models, iron(III)-iodosylarene complex, sulfoxidation, epoxidation, O-atom transfer, catalysis

## Abstract

Previous biomimetic studies clearly proved that equatorial ligands significantly influence the redox potential and thus the stability/reactivity of biologically important oxoiron intermediates; however, no such studies were performed on Fe^III^OIPh species. In this study, the influence of substituted pyridine co-ligands on the reactivity of iron(III)-iodosylbenzene adduct has been investigated in sulfoxidation and epoxidation reactions. Selective oxidation of thioanisole, *cis*-cyclooctene, and *cis*- and *trans*-stilbene in the presence of a catalytic amount of [Fe^II^(PBI)_3_](OTf)_2_ with PhI(OAc)_2_ provide products in good to excellent yields through an Fe^III^OIPh intermediate depending on the co-ligand (4R-Py) used. Several mechanistic studies were performed to gain more insight into the mechanism of oxygen atom transfer (OAT) reactions to support the reactive intermediate and investigate the effect of the equatorial co-ligands. Based on competitive experiments, including a linear free-energy relationship between the relative reaction rates (log*k*_rel_) and the *σ*_p_ (4R-Py) parameters, strong evidence has been observed for the electrophilic character of the reactive species. The presence of the [(PBI)_2_(4R-Py)Fe^III^OIPh]^3+^ intermediates and the effect of the co-ligands was also supported by UV-visible measurements, including the color change from red to green and the hypsochromic shifts in the presence of co-ligands. This is another indication that the title iron(III)-iodosylbenzene adduct is able to oxygenate sulfides and alkenes before it is transformed into the oxoiron form by cleavage of the O−I bond.

## 1. Introduction

In nature, there are many nonheme iron-containing oxidoreductases, which are responsible for important metabolic oxidation processes, including C-H and C=C activation, resulting in hydroxylated, halogenated, or epoxidated products [1,2,3,4,5,6,7,8]. The factors that regulate the ability of iron centers to catalyze these reactions are still obscured. The study of iron cofactors and their role in biological oxidation processes are a key target for biochemists and bio-inorganic chemists as well. The development of synthetic biomimetic systems, including the synthesis and characterization of reactive intermediates, may help to better understand these curious processes [9,10,11,12,13,14,15,16,17]. 

Predicting the correct oxidation state and nature of reactive intermediates in metal-containing oxygenase enzymes and their biomimetics is always a controversial issue. The generation of the putative intermediates can be achieved using different oxidizing agents in one or more steps through the formation of reactive species. Most of the iron-mediated oxidation processes can be interpreted through the high-valence oxoiron(IV, V) intermediates, but the role of their precursors, such as Fe^III^-OX (X = IPh, OH, OR), cannot be ruled out either. It has been shown that the reactivity of high-valent metal-oxo complexes is influenced by various factors, such as the structure and topology of the supporting ligands, the nature of the axial and equatorial co-ligands, and the oxidation and spin state of the metal ion(s) [18,19,20,21,22,23,24,25,26,27,28,29,30]. An increase in oxidation state results in an increase in electrophilicity of the oxoiron species, which significantly reduces the barrier height of C–H activation (69.7 kJ/mol for Fe^IV^=O, 16.8 kJ/mol for Fe^V^=O) [31]. The effect of different ligand environments, including the equatorial and axial co-ligands, on the reactivity of oxoiron(IV) intermediates has been investigated for various oxidation reactions. Previous studies show that the nature of the equatorial ligands plays a decisive role in the redox potential values of iron-oxo complexes [18].

There are only a few examples in the literature where the role of precursor intermediates, such as Fe^III^OIPh, can be clearly demonstrated in C-H activation and oxygen atom transfer reactions [32,33,34,35,36,37,38,39,40,41,42,43,44,45,46]. Well-characterized penta- and hepta-coordinated Fe^III^OIPh intermediates were reported using tetradentate 13-TMC (13-TMC = 1,4,7,10-tetramethyl-1,4,7,10-tetraazacyclotridecane) and hexadentate tpena (tpena = *N*,*N*,*N*′-tris(2-pyridylmethyl)ethylenediamine-*N*′-acetate) ligands [32,33,34]. Based on theoretical studies of (tpena)Fe^III^OIPh-mediated thioanisole sulfoxidation, direct oxygen atom transfer is expected via two mechanisms, namely, bond-cleavage coupled electron transfer (BCCET) and direct oxygen atom transfer (DOT), which are comparable considering their calculated barrier heights [35]. Based on experimental studies of (13-TMC)Fe^III^OIPh-mediated styrene epoxidation, a DOT mechanism was proposed, which is consistent with the theoretical calculation resulting in simultaneous cleavage of the I−O bond and O−C bond formation during the epoxide formation [18,36]. Related to this area, we investigated the reactivity of the in situ formed (PBI)Fe^III^OPh (PBI = 2-(2-pyridyl)benzimidazole) intermediate in oxygen atom transfer reactions towards thioanisole and styrene derivatives [45,46,47]. Based on mechanistic studies, we obtained clear evidence that the stoichiometric and catalytic thioanisole sulfoxidation reactions mediated by the Fe^III^OIPh intermediate proceeds via a DOT mechanism, in contrast to styrene oxygenation, where the epoxidation can be depicted by a nonconcerted ET mechanism [47]. 

**Scheme 1:**
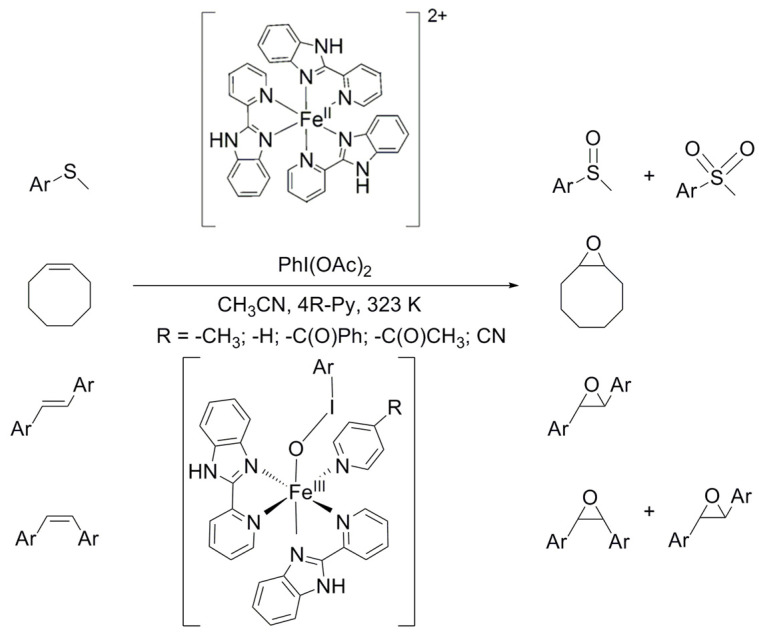
Catalytic sulfoxidation and epoxidation by [Fe^II^(PBI)_3_](OTf)_2_ in the presence of PhI(OAc)_2_ oxidant and 4R-Py co-ligands.

Previous experimental studies have clearly demonstrated that different types of equatorial ligands significantly affect the redox potential and thus the stability/reactivity of oxoiron species; however, no such studies have been performed on Fe^III^OIPh species. The aim of the present work is to investigate the effect of pyridine derivatives as possible co-ligands in our previously studied and published (PBI)Fe^III^OIPh-mediated catalytic epoxidation and sulfoxidation reactions [47] (Scheme 1). 

Based on the previously proposed structure of the (PBI)_2_(Solv)Fe^III^OIPh (Solv = MeCN or H_2_O) intermediate, we expected the steric effect of the two PBI ligands to be minimal; therefore, the effective ligand field is expected to be primarily related to the electronic properties of the equatorial pyridine co-ligands. The reactivities of the new Fe^III^OIPh units and the effect of the co-ligands toward various alkene and thioanisole derivatives have been investigated and are discussed below.

## 2. Results and Discussion

To test the catalytic activity of the previously synthesized and characterized ferrous complex, [Fe^II^(PBI)_3_](OTf)_2_ (**1**) (OTf = CF_3_SO_3_) as catalyst [48], the sulfoxidation reactions of thioanisole and the epoxidation reactions of cis-cyclooctene and cis- and trans-stilbene were investigated using PhI(OAc)_2_ as an oxidant under optimal catalytic conditions (1:100:300 ratio for catalyst:oxidant:substrate) in acetonitrile at 323 K. The advantage of the PhI(OAc)_2_ as a mild oxygen source is that it does not damage the catalysts nor show appreciable reactivity to the substrate [49]. According to our earlier studies, the trace amount of water can cause the steady and slow formation of the more oxidizing PhIO from the PhI(OAc)_2_ [50]. Control experiments showed that no oxidized products were formed in the absence of either the catalyst or the PhI(OAc)_2_. The effect of electron-donating and electron-withdrawing substituents on the pyridine additives as possible co-ligands on the relative reactivity has also been studied in competitive experiments and showed a significant impact on both the catalytic sulfoxidation and epoxidation reactions.

### 2.1. Influence of Equatorial Ligands on the Fe^III^OIPh-Mediated Catalytic Sulfoxidation Reactions

Organic sulfoxides and sulfones are valuable synthetic intermediates that are increasingly used for the production of chemically and biologically active molecules such as agrochemicals, drugs, and synthetic intermediates [51,52]. We have found earlier that the [Fe(PBI)_3_](OTf)_2_ complex catalyzes the efficient oxidation of thioanisole derivatives to appropriate sulfoxides with iodosylbenzene (PhIO), and the metastable iron(III)-iodosylbenzene was chemically generated and kinetically studied in the oxidation of thioanisole substrates [47]. The competition results (including linear free-energy relationships between the relative reaction rates (logk_rel_) and the σ_p_ (4R-PhSMe) parameters with *ρ* = −1.13) suggested that the electrophilicity of the Fe^III^(OIPh) intermediate is crucial to control the nature of the active oxidant form that significantly affects its catalytic activity. Furthermore, based on the small negative *ρ* value, the oxygenation reaction probably follows a DOT mechanism. 

**Figure 1:**
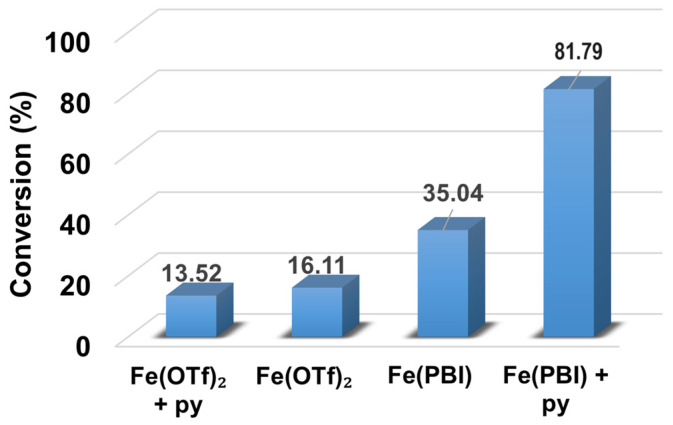
Catalytic oxidation of thioanisole carried out by Fe^II^(OTf)_2_ and [Fe^II^(PBI)_3_](OTf)_2_ with PhI(OAc)_2_ in the presence and absence of pyridine in acetonitrile at 323 K: [Fe^II^(OTf)_2_]_0_ = 1 × 10^−3^ M, [[Fe^II^(PBI)_3_](OTf)_2_]_0_ = 1 × 10^−3^ M, [PhI(OAc)_2_]_0_ = 1 × 10^−1^ M, [thioanisole]_0_ = 3 × 10^−1^ M, [pyridine]_0_ = 1 × 10^−2^ M.

We would like to obtain additional evidence to support the above, using well-chosen additives, para-substituted pyridines (4R-Py, R = -Me, -H, -COC_6_H_5_, -COMe, -CN) as possible equatorial co-ligands. The reactions were carried out under standard catalytic conditions (1:100:300 ratio for [Fe(PBI)_3_](OTf)_2_:PhI(OAc)_2_:PhSMe) in acetonitrile at 323 K. The large excess of substrate was used to minimize over-oxidation of the product. The optimal catalyst:co-ligand ratio is 1 to 10 based on stoichiometric results (see latter sections). In the first round, it can be established that the catalytic activity of the iron salt, [Fe^II^ (CH_3_CN)_4_(OTf)_2_], is negligible (yield: 16.11%). A somewhat smaller but not significant decrease in activity was found for [Fe^II^(py)_4_(OTf)_2_]^2+^ (Fe(II):py = 1:10), with 13.52% yield. The comprehensive screening results for [Fe(PBI)_3_](OTf)_2_ with and without additives are compiled in Table 1. As expected, methyl phenyl sulfoxide (35–89% yields and a small amount of methyl phenyl sulfone (~1–2%) were the only oxidized products without any other byproducts, such as disulfide after 4 h. Notably, a favorable and significant effect could be achieved by the use of pyridine compared to the parent compound (from 35.04 to 81.79%), [Fe(PBI)_3_](OTf)_2_ (Figure 1). Based on these results, the formation of [Fe^II^(py)_4_(OTf)_2_] by replacing PBI with 10 equivalent of py ligands is unlikely during the catalytic process. 

**Figure 2:**
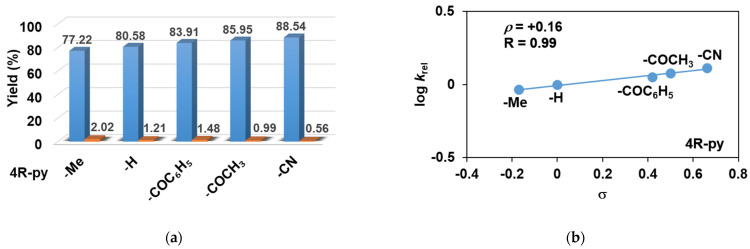
Catalytic oxidation of thioanisole carried out by [Fe^II^(PBI)_3_](OTf)_2_ with PhI(OAc)_2_ and *para*-substituted pyridine additives in acetonitrile at 323 K: (**a**) The calculated conversion (=TON) values for *para*-substituted pyridines. (**b**) Hammett plot of log*k*_rel_ against the *σ*_p_ of *para*-substituted pyridines. [Fe^II^(OTf)_2_]_0_ = 1 × 10^−3^ M [[Fe^II^(PBI)_3_](OTf)_2_]_0_ = 1 × 10^−3^ M, [PhI(OAc)_2_]_0_ = 1 × 10^−1^ M, [thioanisole]_0_ = 3 × 10^−1^ M, [pyridine]_0_ = 1 × 10^−2^ M.

As evident in Table 1, the sulfoxidation reaction was affected by the electronic effects of the substituents on the pyridine additives used. For example, the system containing 4-CN-pyridine resulted in the highest conversion value for the oxidation of thioanisole (88.54%). In contrast, the additive 4-Me-pyridine yielded the lowest conversion, 77.22%. The effect is not significant but can be attributed to the increased electrophilicity of the reactive intermediate due to the electron-withdrawing substituents of the pyridine co-ligand. In addition, the relative rates (*k*_rel_) for the catalytic oxidation of thioanisole in the presence of substituted pyridine (4R-Py, R = -Me, H, -COC_6_H_5_, -COMe and CN) additives were evaluated by monitoring the formation of sulfoxides by GC. Figure 2b depicts a linear correlation (R = 0.99) of log *k*_rel_ (*k*_rel_ = log(*X*_f_/*X*_i_)/log(*Y*_f_/*Y*_i_), where *X*_i_ and *X*_f_ are the initial and final concentration of the PhSMe in the presence of 4R-Py, and Y_i_ and *Y*_f_ are the initial and final concentration of the PhSMe in the presence of Py) versus Hammett σ_p_ substituent constant in the competitive oxidation by [Fe^II^(PBI)_3_](OTf)_2_ catalyst. The slope (*ρ*) of the plot is +0.16, which means that the electron-deficient intermediates are much more reactive than the electron-rich derivatives. Based on this result, a single mechanism with the involvement of an electrophilic oxidant, most likely a [(PBI)_2_(4R-Py)Fe^III^OIPh]^3+^ intermediate, can be proposed. 

Summarizing the results of the competitive experiments, it can be said that the small positive value obtained for the co-ligands (*ρ* = +0.16) and the previous small negative value obtained for the substrates (*ρ* = −0.65 [33]) are consistent with each other and presumably refer to a direct oxygen transfer (DOT) mechanism. A much more significant effect and evidence for the presence of the thioanisole radical cation at 540 nm in the UV-Vis spectrum is expected in the case of an electron-transfer (ET) mechanism [18].

### 2.2. Influence of Equatorial Ligands on the Fe^III^OIPh-Mediated Catalytic Epoxidation Reactions

The epoxidation of olefins to epoxides as intermediates is a fundamental process in organic synthesis, as their stereoselective ring opening with nucleophiles can yield valuable compounds with two adjacent chiral centers [52,53]. Because of this, the investigation of oxygen transfer reactions was also extended to alkene derivatives. For comparison, the epoxidation reactions were carried out under the conditions used for the sulfoxidation reactions (1:100:300 ratio for catalyst:oxidant:substrate in acetonitrile at 323 K). We chose *cis*-cyclohexene and *cis*- and *trans*-stilbene as model substrates. In the first round, it can be established that the catalytic activity of the iron salt, [Fe^II^ (CH_3_CN)_4_(OTf)_2_], is negligible (yields: 9.41–17.76%). A somewhat smaller but not significant decrease in activity was found for [Fe^II^(py)_4_(OTf)_2_]^2+^ (Fe(II):py = 1:10), with 6.31–9.79% yields.

**Figure 3:**
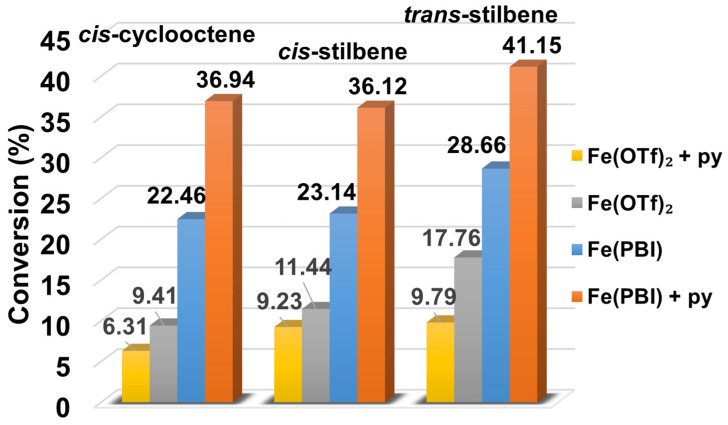
Catalytic oxidation of *cis*-cyclooctene and *cis*- and *trans*-stilbene carried out by Fe^II^(OTf)_2_ and [Fe^II^(PBI)_3_](OTf)_2_ with PhI(OAc)_2_ in the presence and absence of pyridine in acetonitrile at 323 K: [Fe^II^(OTf)_2_]_0_= 1 × 10^−3^ M [[Fe^II^(PBI)_3_](OTf)_2_]_0_ = 1 × 10^−3^ M, [PhI(OAc)_2_]_0_ = 1 × 10^−1^ M, [alkenes]_0_ = 3 × 10^−1^ M, [pyridine]_0_ = 1 × 10^−2^ M.

Based on the preliminary experiments for [Fe^II^(PBI)_3_](OTf)_2_, it can be concluded that the py additive increases the conversion values by approximately 1.4–1.6 times compared to the parent pyridine-free system. Based on the obtained conversion values (=TON), the following order of reactivity can be established for the tested alkenes: *trans*-stilbene (41.15%) > *cis*-cyclooctene (36.94%) > *cis*-stilbene (36.12%). It can also be stated that the values obtained are much smaller than the values obtained for thioanisole (81.79%), which indicates that sulfoxidation is much more favorable than epoxidation in the investigated system (Figure 3 and Table 2, Table 3 and Table 4).

**Figure 4:**
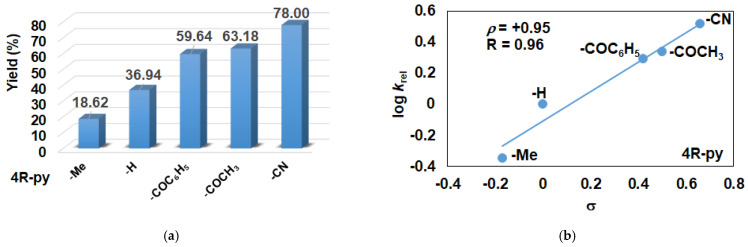
Catalytic oxidation of *cis*-cyclooctene carried out by [Fe^II^(PBI)_3_](OTf)_2_ with PhI(OAc)_2_ and *para*-substituted pyridine additives in acetonitrile at 323 K: (**a**) The calculated conversion values for *para*-substituted pyridines. (**b**) Hammett plot of log*k*_rel_ against the *σ*_p_ of *para*-substituted pyridines. [Fe^II^(OTf)_2_]_0_ = 1 × 10^−3^ M [[Fe^II^(PBI)_3_](OTf)_2_]_0_ = 1 × 10^−3^ M, [PhI(OAc)_2_]_0_ = 1 × 10^−1^ M, [*cis*- cyclooctene]_0_ = 3 × 10^−1^ M, [4R-Py]_0_ = 1 × 10^−2^ M.

**Figure 5:**
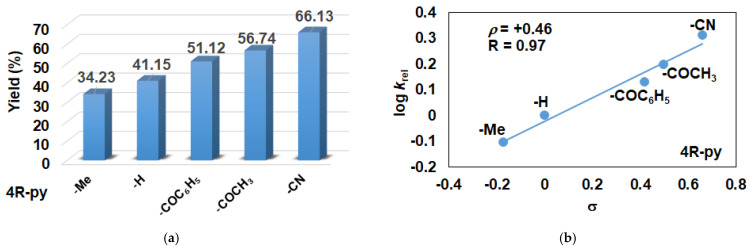
Catalytic oxidation of *trans*-stilbene carried out by [Fe^II^(PBI)_3_](OTf)_2_ with PhI(OAc)_2_ and different *para*-substituted pyridine derivatives in acetonitrile at 323 K: (**a**) The calculated conversion values for para-substituted pyridines. (**b**) Hammett plot of log*k*_rel_ against the *σ*_p_ of para-substituted pyridines. [Fe^II^(OTf)_2_]_0_ = 1 × 10^−3^ M [[Fe^II^(PBI)_3_](OTf)_2_]_0_ = 1 × 10^−3^ M, [PhI(OAc)_2_]_0_ = 1 × 10^−1^ M, [*trans*-stilbene]_0_ = 3 × 10^−1^ M, [4R-Py]_0_ = 1 × 10^−2^ M.

**Figure 6:**
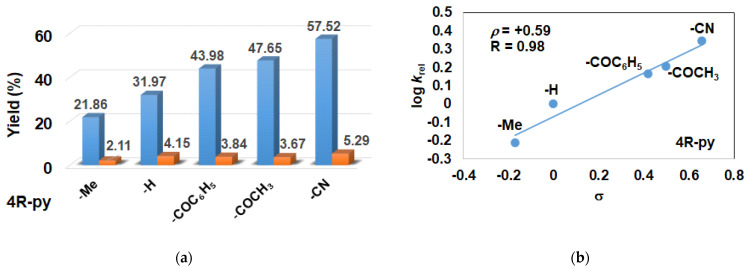
Catalytic oxidation of *cis*-stilbene carried out by [Fe^II^(PBI)_3_](OTf)_2_ with PhI(OAc)_2_ and *para*-substituted pyridine additives in acetonitrile at 323 K: (**a**) The calculated conversion values for *para*-substituted pyridines. (**b**) Hammett plot of log*k*_rel_ against the *σ*_p_ of *para*-substituted pyridines. [Fe^II^(OTf)_2_]_0_ = 1 × 10^−3^ M [[Fe^II^(PBI)_3_](OTf)_2_]_0_ = 1 × 10^−3^ M, [PhI(OAc)_2_]_0_ = 1 × 10^−1^ M, [*cis*-stilbene]_0_ = 3 × 10^−1^ M, [4R-Py]_0_ = 1 × 10^−2^ M.

The effect of the electron-donating and electron-withdrawing substituents of the pyridine additives on the relative reactivity was also studied for the listed alkenes, and a significant effect on the catalytic epoxidation reactions was shown (Table 2, Table 3 and Table 4 and Figure 4a, Figure 5a and Figure 6a). Electron-withdrawing groups such as -CN gave significantly better yields (62.81, 66.13, and 78%) than electron-donating groups such as -Me (23.97, 34.23, and 18.72%) for *cis*-stilbene, *cis*-cyclooctene, and *trans*-stilbene, respectively.

The catalytic effect of *para*-substituted 4R-Py additives as co-ligands relative to that of Py in the epoxidation reactions of the listed alkenes was also investigated (Table 2, Table 3 and Table 4 and Figure 4b, Figure 5b and Figure 6b). Competitive reactions were performed with *para*-substituted pyridine additives to evaluate the effect of electronic factors on the catalytic epoxidation reaction of *cis*-stilbene, *cis*-cyclooctene, and *trans*-stilbene, respectively. The relative rates (*k*_rel_) for the catalytic oxidation of the listed alkenes in the presence of substituted pyridine (4R-Py, R = -Me, -H, -COC_6_H_5_, -COMe and -CN) additives were evaluated by monitoring the formation of the appropriate epoxides using GC. The Hammett treatments of relative reactivities (log *k*_rel_) (*k*_rel_ = log(*X*_f_/*X*_i_)/log(*Y*_f_/*Y*_i_)—where *X*_i_ and *X*_f_ are the initial and final concentration of the appropriate alkene in the presence of 4R-Py, and *Y*_i_ and *Y*_f_ are the initial and final concentration of the appropriate alkene in the presence of Py) versus Hammett σ_p_ substituent constant in the competitive oxidation reactions—gave *ρ* values of +0.95 (Figure 4b), +0.59 (Figure 6b), and +0.46 (Figure 5b) for *cis*-cyclooctene, *cis*-stilbene, and *trans*-stilbene, respectively, which suggests that the behavior of the oxidant (most likely a (PBI)_2_(4R-Py)Fe^III^OIPh) generated from [Fe^II^(PBI)_3_](OTf)_2_ and PhI(OAc)_2_ (PhIO) is electrophilic. These values are much higher compared to that was observed for PhSMe (+0.16). 

*Cis*-stilbene is a model substrate used for investigating the epoxidation mechanism and obtaining mechanism information with the ratio of *cis*- and *trans*-isomers in the stilbene oxide product. The catalytic oxidation of *cis*-stilbene was achieved with ~8% retention of configuration to *cis*-stilbene epoxide and ~92% to *trans*-epoxide, which may be explained by an electron-transfer mechanism with the formation of a short-lived acyclic radical intermediate, or an intermediate carbocation, which led to a partial loss of stereochemistry, affording a mixture of *cis*- and *trans*-epoxides [54]. The oxidation of *trans*-stilbene was achieved with retention of configuration (~100% selectivity), which also establishes that the epoxide configuration is mainly determined by the steric effect. The rotation around the C-C bond is not observed in the oxidation of *trans*-stilbene since the repulsion of the phenyl groups in *trans*-stilbene is much smaller than *cis*-stilbene.

Based on the obtained results, it can be assumed that the oxidation of aromatic alkenes catalyzed by [Fe^II^(PBI)_3_](OTf)_2_ with PhI(OAc)_2_ as an oxidant in the presence of pyridines takes place through the formation of a rate-limiting radical intermediate. 

### 2.3. Catalytic Oxidation of Cis-Cyclooctene Followed by UV-Vis Measurements (Mechanistic Studies)

**Figure 7:**
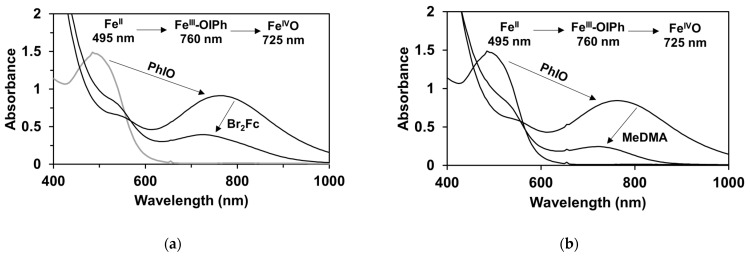
Reaction of the in situ generated Fe^III^-OIPh species with (**a**) 1,1′-dibromoferrocene (Br_2_Fc) and (**b**) with *N*,*N*-dimethylamino-benzene (MeDMA) in acetonitrile at 293 K. [Fe^II^(PBI)_3_](OTf)_2_ = ~0.8 × 10^−3^ M, PhIO = 4.0 × 10^−3^ M, [Br_2_Fc] = 1 × 10^−3^ M, [MeDMA] = 1 × 10^−3^ M, l = 1 cm, V = 2 mL MeCN.

We have previously reported the possible generation of the reactive iron(III)-iodosylbenzene adduct (λ_max_ = 760 nm; ε = 1400 M^−1^ cm^−1^; with S = ½ low-spin state) by the reaction of [Fe^II^(PBI)_3_](OTf)_2_ with an excess of PhIO. Its formation mechanism and composition are currently not clear, but based on preliminary data, the following [(PBI)_2_[(Solvent)Fe^III^(OIPh)]^3+^ formula can be proposed. To this end, the oxidation of [Fe^II^(PBI)_3_](OTf)_2_ with PhI(OAc)_2_ (PhIO) was conducted to probe the nature of the active oxidizing intermediate in the absence of substrates. As shown in Figure 7, the addition of an excess of PhIO resulted in a fast conversion of [Fe^II^(PBI)_3_](OTf)_2_ to a proposed [(PBI)_2_[(Solvent)Fe^III^(OIPh)]^3+^ intermediate. Its reaction with 1,1′-dibromoferrocene (Br_2_Fc) and *N*,*N*-dimethylamino-benzene (MeDMA) in acetonitrile at 293 K resulted in the formation of [(PBI)_2_[(Solvent)Fe^III^(O)]^2+^ in both cases based on its characteristic spectral features ((λ_max_ = 725 nm; ε~400 M^−1^ cm^−1^) (Figure 7a and Figure 7b, respectively). Based on these results, we cannot rule out the possibility that the oxoiron(V) species is formed via heterolytic cleavage of the O-I bond of the iron(III)-iodosylbenzene adducts in a pre-equilibrium process, and this intermediate participates as a reactive element in the oxidation process.

**Figure 8:**
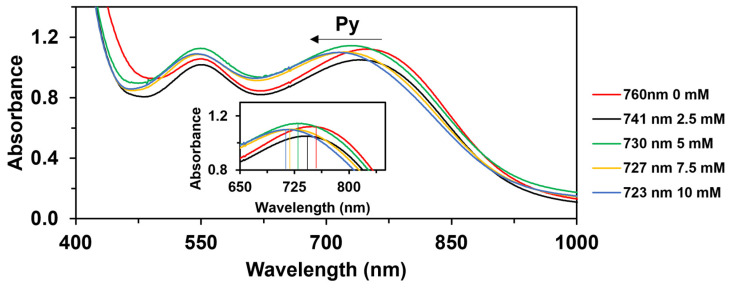
UV-Vis spectral change during the titration of the in situ generated [(PBI)_2_[(Solvent)Fe^III^(OIPh)]^3+^ species with 2.5–10 equivalent of pyridine in acetonitrile at 323 K: [[Fe^II^(PBI)_3_](OTf)_2_]_0_ = 1 × 10^−3^ M, [PhI(OAc)_2_]_0_ = 1 × 10^−3^ M, [pyridine]_0_ = 0.25 − 1 × 10^−2^ M.

However, when the stoichiometric epoxidation and sulfoxidation reactions were investigated with an excess of PhI, nearly two-fold reaction rates were observed from the saturation curves, suggesting that the Fe^III^(IOPh)-mediated reaction is more favored compared to the oxoiron(V) species and that the equilibrium is shifted toward the Fe^IlI^(OIPh) species [47]. When the reaction of [Fe^II^(PBI)_3_](OTf)_2_ (1 mM) with excess of PhIO(Ac)_2_ (50 mM) was repeated in the presence of cyclooctene (300 mM), the UV-Vis spectra show the clean formation and decay of the proposed [(PBI)_2_[(Solvent)Fe^III^(OIPh)]^3+^ intermediate by its known λ_max_ at 760 nm. If the proposed structure is correct, the coordinated solvent molecule can be replaced by substituted pyridines in an equilibrium process, and the effect of electron-donor and electron-withdrawing substituents can be investigated. The optimal amount (10 equiv.) of the pyridine co-ligand for the complete formation of the [(PBI)_2_[(Py)Fe^III^(OIPh)]^3+^ intermediate was determined by titrating the in situ generated [(PBI)_2_[(Solvent)Fe^III^(OIPh)]^3+^ with pyridine (Figure 8). The equilibrium process was indicated by the shift of the λ_max_ of the [(PBI)_2_[(Solvent)Fe^III^(OIPh)]^3+^ adduct. The complete formation of [(PBI)_2_[(4R-Py)Fe^III^(OIPh)]^3+^ intermediates requires 8–10 equivalents of pyridines, and the adducts were also stable even in the presence of a large excess of pyridins (20–30 equivalents). Based on these results, it can be said that under the conditions used, the formation of [Fe^II^(Py)_4_(OTf)_2_] and its iron(III)-iodosylbenzene adduct can be ruled out. This was also supported by the UV-Vis spectra of the [Fe^II^(Py)_4_(OTf)_2_] and [Fe^II^(MeCN)_4_(OTf)_2_]-containing catalytic systems, where the formation of the iron(III)-iodosylbenzene adducts can not be observed.

**Figure 9:**
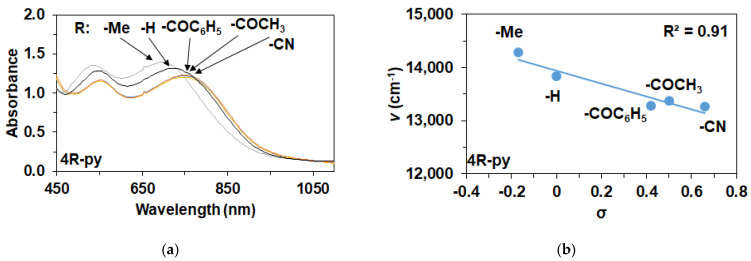
Catalytic oxidation of *cis*-cyclooctene carried out by [Fe^II^(PBI)_3_](OTf)_2_ with PhI(OAc)_2_ and *para*-substituted pyridine additives in acetonitrile at 298 K: (**a**) UV-Vis spectra of [(PBI)_2_[(4R-Py)Fe^III^(OIPh)]^3+^ intermediates. [[Fe^II^(PBI)_3_](OTf)_2_]_0_ = 1 × 10^−3^ M, [PhI(OAc)_2_]_0_ = 5 × 10^−2^ M, [*cis*-cyclooctene]_0_ = 3 × 10^−1^ M, [4R-Py]_0_ = 1 × 10^−2^ M. (**b**) ν vs. σ values for *para*-substituted pyridines.

When the reactions were reacted in the presence of 10 equivalents of pyridines, a hypsochromic, so-called blue shift was observed (Table 5 and Figure 9a). 

**Figure 10:**
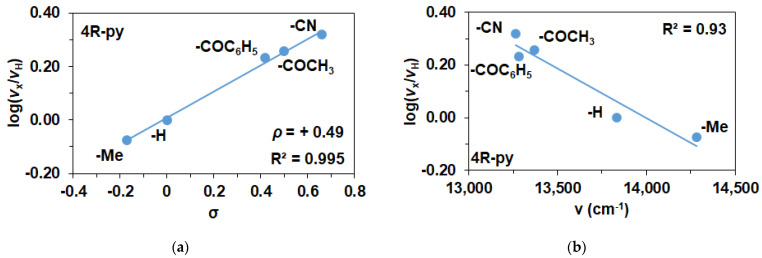
Catalytic oxidation of *cis*-cyclooctene carried out by [Fe^II^(PBI)_3_](OTf)_2_ with PhI(OAc)_2_ and *para*-substituted pyridine additives in acetonitrile at 298 K: (**a**) Hammett plot of log*(v_X_/v_H_)* against the *σ* of *para*-substituted pyridines. (**b**) log(*v*_X_/*v*_H_) vs. ν values for *para*-substituted pyridines. [[Fe^II^(PBI)_3_](OTf)_2_]_0_ = 1 × 10^−3^ M, [PhI(OAc)_2_]_0_ = 5 × 10^−2^ M, [*cis*-cyclooctene]_0_ = 3 × 10^−1^ M, [4R-Py]_0_ = 1 × 10^−2^ M.

The largest shift (60 nm) was obtained for the 4Me-Py, while the smallest one was obtained for the 4CN-Py (6 nm). These results support the coordination of 4R-Py molecules as equatorial co-ligands to the iron center. More importantly, a linear correlation was found between the energy of the characteristic absorption bands (λ_max_^−1^ at 700–760 nm) and the Hammett substituent constants (σ_p_), indicating that the observed shift in the absorption bands can be assigned indirectly to the electronic effect of the co-ligands and the electrophilicity of the active intermediate (Figure 9b). We have also found that the decay rate of the [(PBI)_2_[(4R-Py)Fe^III^(OIPh)]^3+^ species is affected by *cis*-cyclooctene and depends on the pyridine co-ligands. The Hammett treatments of relative reactivities (log *k*_rel_) (*k*_rel_ = *V*_X_/*V*_H_), where *V*_X_ and *V*_H_ are the initial reaction rates calculated from the decrease in the absorbance in the 700–760 nm region) versus Hammett σ_p_ substituent constant (and ν) in the competitive oxidation reactions gave *ρ* values of +0.49 (Figure 10a,b). This value is consistent with the results of the competitive measurements carried out by the GC method.

## 3. Materials and Methods

All materials, including PBI ligands and substrates, were obtained from Aldrich Chemical Co. and used without further purification unless otherwise indicated. Solvents were dried according to published procedures, distilled, and stored under argon. [Fe(PBI)_3_](OTf)_2_ was synthesized according to literature methods [48]. IR spectra were recorded using a Thermo Nicolet Avatar 330 FT-IR instrument (Thermo Nicolet Corporation, Madison, WI, USA). The UV-Visible spectra were recorded on an Agilent 8453 (Budapest, Hungary) diode-array spectrophotometer using quartz cells. GC and GC-MS analyses were carried out on a Agilent 7820 A (Budapest, Hungary) equipped with flame ionization detector and a 30 m Equity-1 column and Shimadzu QP2010SE (Budapest, Hungary) equipped with a secondary electron multiplier detector with conversion dynode and a 30 m HP5MS column, respectively. 

*Catalytic reactions by UV-Vis spectroscopy*: [Fe^II^(PBI)_3_](OTf)_2_ (1 × 10^−3^ M) was dissolved in acetonitrile (3 mL), and different *para*-substituted pyridines (1 × 10^−2^ M) and (diacetoxyiodo)benzene (5 × 10^−2^ M) and then *cis*-cyclooctene (3 × 10^−1^ M) were added to the solution. The reactions were carried out at 298 K. The initial absorbance of [(PBI)_2_(4R-Py)Fe^III^OIPh]^3+^ was measured, and its reaction was followed by a decrease in absorbance at 723–760 nm. Initial reaction rates were calculated using Biochemical Analysis Software for Agilent 8453 ChemStation software (2002) (https://www.agilent.com/en/product/molecular-spectroscopy/uv-vis-uv-vis-nir-spectroscopy/uv-vis-uv-vis-nir-software/uv-vis-chemstation-software, accessed on 18 December 2023) at a conversion value of 30%. 

*Catalytic oxidations:* [Fe^II^(PBI)_3_](OTf)_2_ (1 × 10^−3^ M) was dissolved in acetonitrile (3 mL), and (diacetoxyiodo)benzene (1 × 10^−1^ M), substrate (3 × 10^−1^ M) (thioanisole, *cis*-cyclooctene, *cis*-stilbene, and *trans*-stilbene), and *para*-substituted pyridines (1 × 10^−2^ M) were added to the solution. Bromobenzene (1 × 10^−1^ M) was added to the solution as an internal standard. The mixture was stirred for 4 h at 323 K, the products were identified by GC-MS, and the yields were determined by GC. 

*Products of thioanisole oxidation:*

Methyl phenyl sulfoxide: (*m*/*z*) relative intensity 140.00 (M^+^, 77.54); 125.00 (100); 97.00 (89.79); 94.05 (22.97); 91.05 (10.37); 78.05 (12.95); 77.00 (77.28); 74.00 (12.97); 69.00 (10.66); 65.00 (26.06); 63.00 (11.18); 53.05 (16.08); 51.00 (84.10); 50.05 (35.77); 45.00 (19.61); 39.00 (20.09). 

Methyl phenyl sulfone: (*m*/*z*) relative intensity 155.90 (M^+^, 11.48); 140.95 (15.73); 97.00 (12.72); 94.05 (25.72); 78.00 (10.11); 77.00 (100); 65.05 (20.32); 51.00 (61.48); 50.05 (20.94); 39.05 (15.09).

*Product of cis-cyclooctene oxidation*:

9-Oxabicyclo [6.1.0]nonane: (*m*/*z*) relative intensity 126.10 (M^+^, 19.07); 98.15 (13.20); 97.00 (10.58); 83.10 (15.90); 82.05 (10.83); 79.05 (9.26); 77.00 (9.99); 70.05 (10.65); 69.05 (13.45); 67.00 (44.91); 57.10 (28.88); 56.10 (18.19);55.10 (85.68); 54.10 (24.35); 53.10 (12.53); 51.05 (10.87); 43.10 (18.58); 42.00 (29.09); 41.05 (100); 40.05 (13.26); 39.05 (81.24).

*Products of cis-stilbene oxidation*:

2,3-diphenyloxirane: (*m*/*z*) relative intensity 196.10 (M^+^, 27.46); 195.15 (35.40); 178.05 (19.45); 168.05 (18.42); 167.05 (69.11); 166.15 (12.67); 165.05 (28.99); 152.05 (25.04); 105.15 (23.12); 91.05 (14.46); 90.10 (77.06); 89.00 (100); 77.05 (43.94); 65.05 (12.07); 64.10 (19.47); 63.00 (38.49); 62.00 (13.53); 51.10 (42.21); 50.05 (21.14); 39.05 (28.74).

*trans*-1,2-diphenylethylene oxide: (*m*/*z*) relative intensity 196.00 (M^+^, 26.58); 195.05 (35.66); 180.00 (22.67); 179.05 (24.42); 178.05 (38.77); 168.00 (19.47); 167.05 (74.51); 166.05 (12.19); 165.00 (32.31); 153.00 (10.30); 152.05 (22.57); 105.05 (31.65); 91.05 (21.70); 90.05 (71.10); 89.10 (100); 78.10 (10.96); 77.05 (55.81); 76.05 (12.61); 65.10 (13.23); 64.00 (20.16); 63.10 (43.15); 62.10 (13.55); 52.10 (10.17); 51.05 (55.96); 50.00 (19.23); 39.05 (29.02).

*Product of trans-stilbene oxidation*:

*trans*-1,2-diphenylethylene oxide: (*m*/*z*) relative intensity 196.00 (M^+^, 31.96); 195.05 (40.85); 179.05 (13.64); 178.05 (28.97); 168.05 (19.03); 167.05 (82.09); 166.05 (11.62); 165.05 (16.85); 152.05 (20.29); 105.05 (21.33); 91.05 (15.68); 90.05 (79.24); 89.05 (100); 77.05 (42.40); 64.05 (14.43); 63.05 (35.14); 51.05 (35.71); 50.10 (13.31); 39.05 (19.75).

## 4. Conclusions

**Scheme 2:**
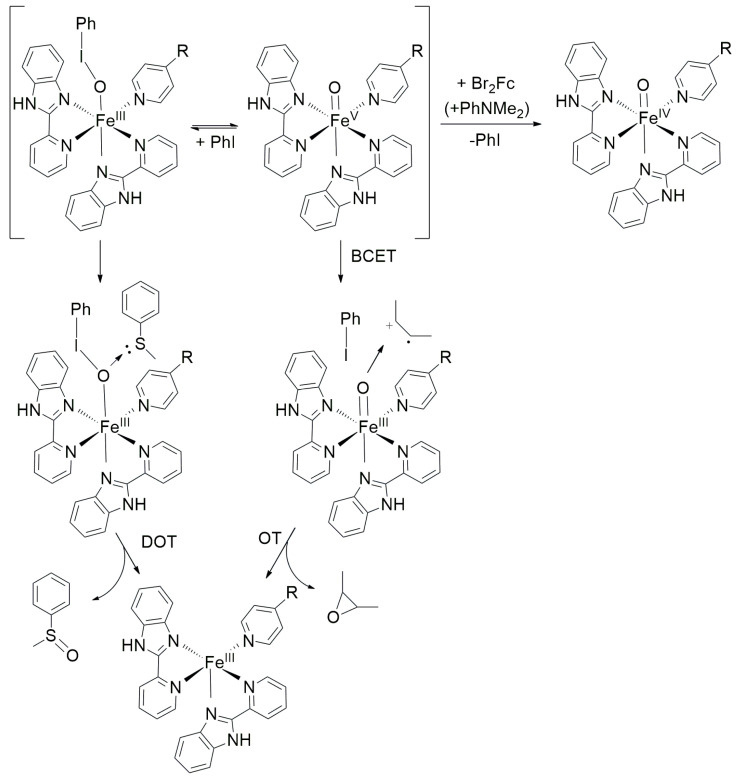
Proposed mechanistic pathways for the iron(III)-iodosylarene ([(PBI)_2_[(4R-Py)Fe^III^(OIPh)]^3+^)-mediated sulfoxidation and epoxidation reactions.

In summary, we reported the catalytic oxidation of thioanisole and various alkene (*cis*-cyclooctene and stilbenes) derivatives using a non-heme complex, Fe^II^(PBI)_3_ as a catalyst and PhI(OAc)_2_ as an oxidant in the presence of pyridine additives. Several mechanistic studies were performed to gain more insight into the mechanism of oxygen atom transfer reactions, support the reactive intermediate, and investigate the effect of the co-ligands. Based on competitive experiments, we established that pyridine additives have a significant effect on the catalytic activity in oxygen transfer processes, probably via their coordination as equatorial co-ligands to the iron center. The greatest increase in reactivity was achieved in the case of pyridines containing an electron-withdrawing group in both epoxidation and sulfoxidation reactions, suggesting the formation of an electrophilic [(PBI)_2_(4R-Py)Fe^III^OIPh]^3+^ intermediate as key oxidant. The results of UV-Vis measurements, including the known characteristic λ_max_ values around 760 nm, are consistent with the formation and decay of the proposed intermediate [(PBI)_2_[(Solvent)Fe^III^(OIPh)]^3+^ during the oxidation reactions. The replacement of the solvent molecule with pyridines resulted in the formation of the appropriate [(PBI)_2_[(4R-Py)Fe^III^(OIPh)]^3+^ species, and the wavelengths of their absorptions decreased in the following order: 754 nm (R = -CN) > 753 (-COMe) > 748 nm (-COPh > 723 (H) > 700 nm (-Me). More importantly, a linear correlation was found between the energy of the characteristic absorption bands (λ_max_^−1^ at 700–760 nm) and the Hammett substituent constants (σ_p_), indicating that the observed hypsochromic shift (blue shift) in the absorption bands can be assigned indirectly to the electronic effect of the co-ligands and the electrophilicity of the active intermediate (Figure 7b). Compared to the parent intermediate ([(PBI)_2_[(Solvent)Fe^III^(OIPh)]^3+^, [(PBI)_2_[(4CN-Py)Fe^III^(OIPh)]^3+^ exhibits enhanced reactivity in both the oxidation of thioanisole and alkenes. Our obtained results are consistent with our previous results, according to which the reaction of thioanisole can be interpreted according to the DOT mechanism, while the oxidation of alkenes can be interpreted according to the ET mechanism (Scheme 2). These results provide further examples of metal–oxidant adducts that can transfer their oxygen atom to organic substrates prior to the conversion into metal oxo species.

## Figures and Tables

**Table 1:** Catalytic oxidation of thioanisole carried out by [Fe^II^(PBI)_3_](OTf)_2_ with PhI(OAc)_2_ and various *para*-substituted pyridine additives in acetonitrile at 323 K ^1^.

Entry	Coligand	Conversion (%) ^2,3^	Products Yields (%) ^2^		Selectivity (%) PhS(O)Me	TOF (1/h) ^4^	*k*_rel_ ^5^
			PhS(O)Me	PhS(O)_2_Me			
1	-	35.04	34.96	0.08	99.7	8.75	-
2	4-Me-Py	79.24	77.22	2.02	97.45	19.81	0.92
3	Py	81.79	80.58	1.21	98.52	20.45	1
4	4-COC_6_H_5_-Py	85.39	83.91	1.48	98.26	21.35	1.13
5	4-COCH_3_-Py	86.94	85.95	0.99	98.86	21.74	1.20
6	4-CN-Py	89.10	88.54	0.56	99.37	22.28	1.30

^1^ Reaction conditions: See experimental section. ^2^ The product was identified by GC-MS, and the yields (based on oxidant) were determined by GC using bromobenzene as internal standard. ^3^ Conversion = TON ([Substrate]_0_/[1]_0_). ^4^ TON/h. ^5^ k_rel_ = log(X_f_/X_i_)/log(Y_f_/Y_i_), where X_i_ and X_f_ are the initial and final concentration of PhSMe in the presence of 4R-Py, and Y_i_ and Y_f_ are the initial and final concentration of PhSMe in the presence of Py. Reaction conditions: [[Fe^II^(PBI)_3_](OTf)_2_]_0_ = 1 × 10^−3^ M, [PhI(OAc)_2_]_0_ = 1 × 10^−1^ M, [PhSMe]_0_ = 3 × 10^−1^ M, [4R-Py]_0_ = 1 × 10^−2^ M.

**Table 2:** Catalytic oxidation of *cis*-cyclooctene carried out by [Fe^II^(PBI)_3_](OTf)_2_ with PhI(OAc)_2_ and *para*-substituted pyridine additives in acetonitrile at 323 K ^1^.

Entry	Co-Ligand	Yield (%) ^2^ Epoxide	TON ^3^	TOF (1/h) ^4^	*k*_rel_ ^5^
1	-	22.46	22.46	5.62	-
2	4-Me-Py	18.62	18.62	4.65	0.44
3	Py	36.94	36.94	9.24	1
4	4-COC_6_H_5_-Py	59.64	59.64	14.91	1.97
5	4-COCH_3_-Py	63.18	63.18	15.79	2.17
6	4-CN-Py	78.00	78.00	19.50	3.28

^1^ Reaction conditions: See experimental section. ^2^ The product was identified by GC-MS, and the yields (based on oxidant) were determined by GC using bromobenzene as internal standard. ^3^ Conversion = TON ([Substrate]_0_/[1]_0_). ^4^ TON/h. ^5^ *k*_rel_ = log(*X*_f_/*X*_i_)/log(*Y*_f_/*Y*_i_), where *X*_i_ and *X*_f_ are the initial and final concentration of the *cis*-cyclooctene in the presence of 4R-Py, and *Y*_i_ and *Y*_f_ are the initial and final concentration of the *cis*-cyclooctene in the presence of Py. Reaction conditions: [[Fe^II^(PBI)_3_](OTf)_2_]_0_ = 1 × 10^−3^ M, [PhI(OAc)_2_]_0_ = 1 × 10^−1^ M, [*cis*-cyclooctene]_0_ = 3 × 10^−1^ M, [4R-Py]_0_ = 1 × 10^−2^ M.

**Table 3:** Catalytic oxidation of *trans*-stilbene carried out by [Fe^II^(PBI)_3_](OTf)_2_ with PhI(OAc)_2_ and *para*-substituted pyridine additives in acetonitrile at 323 K ^1^.

Entry	Co-Ligand	Yield (%) ^2^ *trans*-Epoxide	TON ^3^	TOF (1/h) ^4^	*k*_rel_ ^5^
1	-	36.66	36.66	9.17	-
2	4-Me-Py	34.23	34.23	8.55	0.79
3	Py	41.15	41.15	10.29	1
4	4-COC_6_H_5_-Py	51.12	51.12	12.78	1.35
5	4-COCH_3_-Py	56.74	56.74	14.19	1.58
6	4-CN-Py	66.13	66.13	16.53	2.04

^1^ Reaction conditions: See experimental section. ^2^ The product was identified by GC-MS, and the yields (based on oxidant) were determined by GC using bromobenzene as internal standard. ^3^ Conversion = TON ([Substrate]_0_/[1]_0_). ^4^ TON/h. ^5^ *k*_rel_ = log(*X*_f_/*X*_i_)/log(*Y*_f_/*Y*_i_), where *X*_i_ and *X*_f_ are the initial and final concentration of the *trans*-stilbene in the presence of 4R-Py, and *Y*_i_ and *Y*_f_ are the initial and final concentration of the *trans*-stilbene in the presence of Py. Reaction conditions: [[Fe^II^(PBI)_3_](OTf)_2_]_0_ = 1 × 10^−3^ M, [PhI(OAc)_2_]_0_ = 1 × 10^−1^ M, [*trans*-stilbene]_0_ = 3 × 10^−1^ M, [4R-Py]_0_ = 1 × 10^−2^ M.

**Table 4:** Catalytic oxidation of *cis*-stilbene carried out by [Fe^II^(PBI)_3_](OTf)_2_ with PhI(OAc)_2_ and *para*-substituted pyridine additives in acetonitrile at 323 K ^1^.

Entry	Co-Ligand	Conv. (%) ^2,3^	Products Yields (%) ^2^		Selectivity (%) *trans*-Stilbene Epoxide	TOF (1/h) ^4^	*k*_rel_ ^5^
			*cis*-Stilbene Epoxide	*trans*-Stilbene Epoxide			
1	-	24.14	3.18	19.96	81.76	5.78	-
2	4-Me-Py	23.97	2.11	21.86	91.19	5.99	0.61
3	Py	36.12	4.15	31.97	88.51	9.3	1
4	4-COC_6_H_5_-Py	47.82	3.84	43.98	91.96	11.95	1.45
5	4-COCH_3_-Py	51.32	3.67	47.65	92.85	12.83	1.60
6	4-CN-Py	62.81	5.29	57.52	91.58	15.70	2.20

^1^ Reaction conditions: See experimental section. ^2^ The product was identified by GC-MS, and the yields (based on oxidant) were determined by GC using bromobenzene as internal standard. ^3^ Conversion = TON ([Substrate]_0_/[1]_0_). ^4^ TON/h. ^5^ *k*_rel_ = log(*X*_f_/*X*_i_)/log(*Y*_f_/*Y*_i_), where *X*_i_ and *X*_f_ are the initial and final concentration of the *cis*-stilbene in the presence of 4R-Py, and *Y*_i_ and *Y*_f_ are the initial and final concentration of the *cis*-stilbene in the presence of Py. Reaction conditions: [[Fe^II^(PBI)_3_](OTf)_2_]_0_ = 1 × 10^−3^ M, [PhI(OAc)_2_]_0_ = 1 × 10^−1^ M, [*cis*-stylbene]_0_ = 3 × 10^−1^ M, [4R-Py]_0_ = 1 × 10^−2^ M.

**Table 5:** Catalytic oxidation of *cis*-scyclooctene carried out by [Fe^II^(PBI)_3_](OTf)_2_ with PhI(OAc)_2_ and *para*-substituted pyridine additives in acetonitrile at 298 K. [[Fe^II^(PBI)_3_](OTf)_2_]_0_ = 1 × 10^−3^ M, [PhI(OAc)_2_]_0_ = 5 × 10^−2^ M, [*cis*-cyclooctene]_0_ = 3 × 10^−1^ M, [4R-Py]_0_ = 1 × 10^−2^ M.

Entry	Coligand	λ (nm)	ν (cm^−1^)	σ	*v*_0_ (10^−4^ Ms^−1^) ^1^	log(*v_X_/v_H_*)
1	-	760	13,158	-	-	-
2	4-Me-Py	700	14,286	−0.17	1.26	−0.076
3	Py	723	13,831	0	1.5	0.000
4	4-COC_6_H_5_-Py	748	13,369	0.42	2.56	0.232
5	4-COCH_3_-Py	753	13,280	0.5	2.72	0.258
6	4-CN-Py	754	13,263	0.66	3.12	0.318

^1^ Initial reaction rate.

## Data Availability

Data are contained within the article.
